# Tissue Reaction to Low-Density Polyacrylamide Gel as a Carrier for Microimplants in the Adipose Fin of Rainbow Trout

**DOI:** 10.3390/gels9080629

**Published:** 2023-08-05

**Authors:** Ekaterina Borvinskaya, Svetlana Matrosova, Irina Sukhovskaya, Polina Drozdova, Evgeniy Titov, Inna Anikienko, Yulia Lubyaga, Anton Gurkov, Maxim Timofeyev

**Affiliations:** 1 Institute of Biology, Irkutsk State University, 664025 Irkutsk, Russia; 2 Institute of Biology, Ecology and Agricultural Technologies of the Petrozavodsk State University, 185640 Petrozavodsk, Russia; 3 Institute of Biology, Karelian Research Centre of Russian Academy of Sciences, 185000 Petrozavodsk, Russia; 4 Baikal Research Centre, 664025 Irkutsk, Russia; 5 East Siberian Institute of Medical and Ecological Research, 665827 Angarsk, Russia; 6 Department of Animal Morphology and Veterinary Sanitation, Irkutsk State Agrarian University n.a. A.A. Ezhevsky, 664038 Molodezhniy, Russia

**Keywords:** polyacrylamide, hydrogel, non−biodegradable, injectable implants, fish, rainbow trout, histology

## Abstract

The implantation of optical sensors is a promising method for monitoring physiological parameters of organisms in vivo. For this, suitable hydrogels are required that can provide a biocompatible interface with the organism’s tissues. Amorphous hydrogel is advantageous for administration in animal organs due to its ease of injection compared to resilient analogs. In this study, we investigated the applicability of a semi-liquid 2.5% polyacrylamide hydrogel (PAAH) as a scaffold for fluorescent polyelectrolyte microcapsules (PMs) in rainbow trout. The hydrogel was injected subcutaneously into the adipose fin, which is a small, highly translucent fold of skin in salmonids that is convenient for implanting optical sensors. Using histological methods, we compared tissue organization and in vivo stability of the applied hydrogel at the injection site after administration of uncoated PMs or PMs coated with 2.5% PAAH (PMs-PAAH) for a period of 3 to 14 days. Our results showed that the introduction of PMs into the gel did not have a masking effect, as they were recognized, engulfed, and carried away by phagocytes from the injection site. However, both PMs and PMs-PAAH were found to provoke chronic inflammation at the injection site, although according to cytokine expression in the fish spleen, the irritating effect was local and did not affect the systemic immunity of the fish. Therefore, our study suggests low applicability of 2.5% polyacrylamide as a scaffold for injectable sensors within a timeframe of days.

## 1. Introduction

One of the most promising applications of hydrogels is building various implantable devices, where hydrogels provide a biocompatible interface with the organism’s tissues [1]. Among the new possibilities that can be opened by implantable devices, continuous real−time monitoring of the organism’s internal parameters is one of the most important since it can revolutionize medical and veterinary diagnostics by dramatically amplifying the amount of available physiological data [2]. In the case of implantable sensors, high biocompatibility is particularly critical due to the need to keep the sensor in contact with the interstitial fluids as long as possible by masking it from the immune system, and hydrogels are the most promising candidates to provide such a level of biocompatibility [1,3]. Furthermore, the three−dimensional polymer network of hydrogels is able to hold an impressive amount of water, allowing biological molecules to diffuse into the gel and be measured by the sensitive component within the sensor.

However, implantation of relatively rigid sensory hydrogels requires sophisticated devices or thick transponder needles [4,5,6,7]. At the same time, some semi−liquid hydrogels can serve as scaffolds for injectable sensors, which can be easily and minimally invasively introduced into tissues using a conventional syringe [8,9]. In this case, electronics−based devices are not generally applicable due to their size, but such sensors can use alternative sensitive components, mostly fluorescent and phosphorescent molecular probes or nano−/microsized complex structures for tracking ions, oxygen, metabolites, hormones and drugs [6,10,11,12]. These sensitive components usually indicate the changes in the concentration of the molecule of interest via changes in the form of their luminescence spectrum or in the phosphorescence life−time.

Veterinary medicine is a field in which the application of implantable sensors can have the greatest effect. For example, fish farming technologies do not allow the regular sampling of blood from each individual for animal health diagnostics. Furthermore, each blood sampling is a stressful procedure and increases the risk of fish infection due to tegument damage [13,14]. On the contrary, tracking physiological parameters with implanted sensors would only require an initial puncture of fish skin. The automation of the light−based measurements on fish farms also seems technologically feasible and could provide effective health monitoring of the cultured populations [5].

We have recently shown that in salmonids, one of the top three groups of farmed fish worldwide [15], the highly translucent adipose fin can be conveniently used for the application of implanted optical sensors [16]. In particular, implantation into the adipose fin of rainbow trout *Oncorhynchus mykiss* resulted in over 15−fold more intense fluorescence from the sensor compared to fish skin. However, implantation of the used resilient 13% polyacrylamide hydrogels (PAAH) into the adipose fin required staff training and a multi−step injection procedure, making it difficult to control sterility. In turn, the advantage of semi−liquid hydrogels, such as 2.5% polyacrylamide gel−sols, is that they can be easily and minimally invasively injected into tissues using a conventional syringe [8]. Previously, we applied such an injectable hydrogel as the scaffold for polyelectrolyte microcapsules with a fluorescent molecular pH probe in zebrafish *Danio rerio* [17]. The sensor kept the sensitivity to extracellular pH for at least two days, but later we observed an intense immune response and destruction of the hydrogel carrier. As the rainbow trout adipose fin has a much lower cell and capillary density than the fish muscles and other metabolically active tissues, we hypothesized that the immune response in this organ might be less intense, or at least delayed.

In the current study, we further investigated the effect of subcutaneous injection of 2.5% polyacrylamide on the fish immune system, including assessing its applicability as a scaffold for sensory gel. Our primary objectives were to evaluate the masking effect of the polyacrylamide coating of PMs on immune cells and to assess the stability of the applied hydrogel at the injection site. Polyacrylamide was chosen for this study due to its widespread availability in biological laboratories worldwide and its relatively high stability and histocompatibility [18,19,20,21]. To ensure a realistic assessment of the performance of the material in a living organism, taking into account the dynamic interactions between the material and the surrounding tissues, an in vivo test was carried out with subcutaneous injection of PMs−PAAH into the adipose fin of rainbow trout, followed by histological analysis of the injection site. The usefulness of the hydrogel as a first line of defense against the fluorophore−carrying microcapsules over a two−week period was assessed in comparison with the effect of injecting saline and free polyelectrolyte microcapsules (PMs). The systemic effect of the injection on fish health was assessed by measuring pro−inflammatory gene expression in the trout spleen. As our study was limited to evaluating the mechanical and biological performance of the hydrogel in vivo, the experimental design did not include the investigation of hydrogel nanostructures or chemical transformations.

## 2. Results

### 2.1. Immune Genes Expression in the Spleen

**Figure 1:**
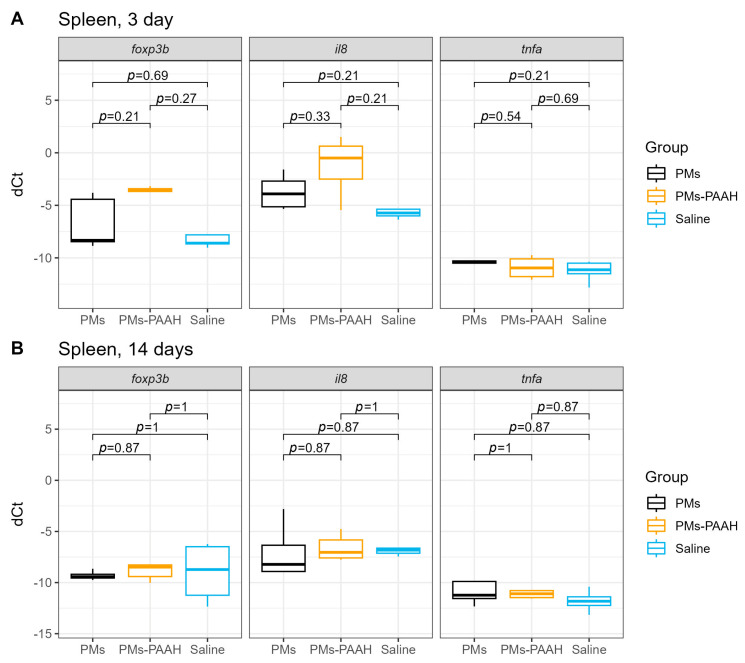
Expression of immune response genes in the trout spleen after subcutaneous injection of PMs, PMs−PAAH, and saline into the adipose fin. (**A**) 3 days after injection; (**B**) 14 days after injection.

Despite the microvolumes used, the injection procedure and the potentially irritating components of the foreign material could have not only local but also systemic adverse effects on the fish organism. The systemic effect of the hydrogel injection procedure was monitored by measuring the expression of pro−inflammatory cytokines, namely phagocyte activator IL8, macrophage−associated cytokine TNFa, and FOXP3b, produced by Treg−like cells in the trout spleen, which is an important immunocompetent organ of fish. On the third day after injection, a higher expression of *foxp3b* and the *il8* genes (statistically insignificant) was observed in the spleen of fish treated with polymeric microcapsules in polyacrylamide gel (PMs−PAAH), compared to the groups that received saline and uncoated PMs (Figure 1A). In turn, on the 14th day after the injection, the level of expression of immune−related genes was similar in all experimental groups of fish (Figure 1B), which indicates the absence of late immune response to microdoses of the polymeric sensor at the organismal level.

### 2.2. Histological Examination

**Figure 2:**
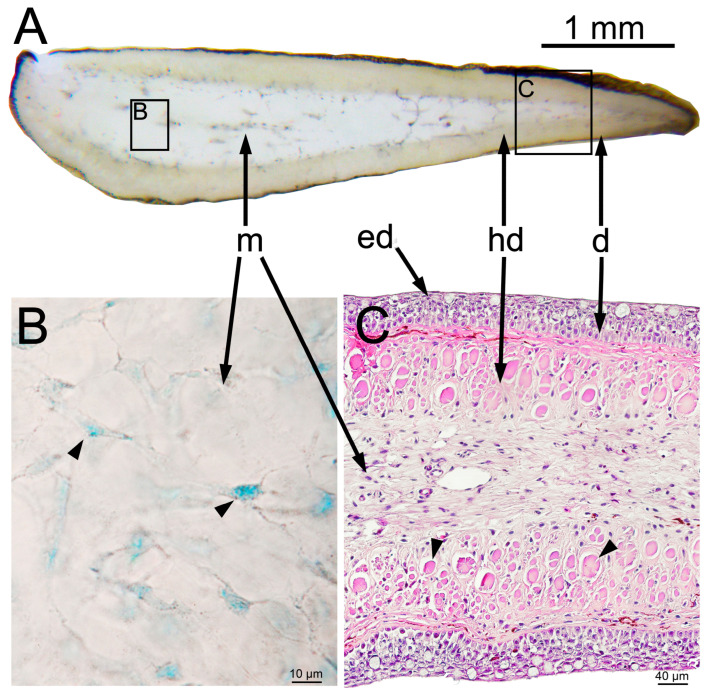
Normal architecture of the adipose fin in juvenile rainbow trout. (**A**) Macrophotograph of a transverse section of an unfixed adipose fin; (**B**) Microscopy of an unfixed section of collagen matrix stained with methylene blue. Arrows point to rare stellate fibroblasts located among amorphous non−cellular substances; (**C**) Histological transverse section of adipose fin (H&E stain). Arrowheads indicate collagen macrofibrils (actinotrichia). Letter designations: ed—epidermis, d—dermis, hd—hypodermis, m—collagen matrix.

To study the processes in the tissue surrounding the injected material, we compared microsection images of intact adipose fin and the site of injection of saline, free PMs or PMs−PAAH. Outside the injection site, the adipose fin morphology was normal and similar to that previously described for other salmonids [22,23]. This organ is a small fold of skin that serves as a vane to track water currents, and has a rather primitive anatomy compared to other fish fins [24,25]. In salmonids, it lacks the endoskeleton, tendons, and musculature for active movement, and is primarily composed of a fleshy core from a connective matrix (Figure 2) [22,23,26].

**Figure 3:**
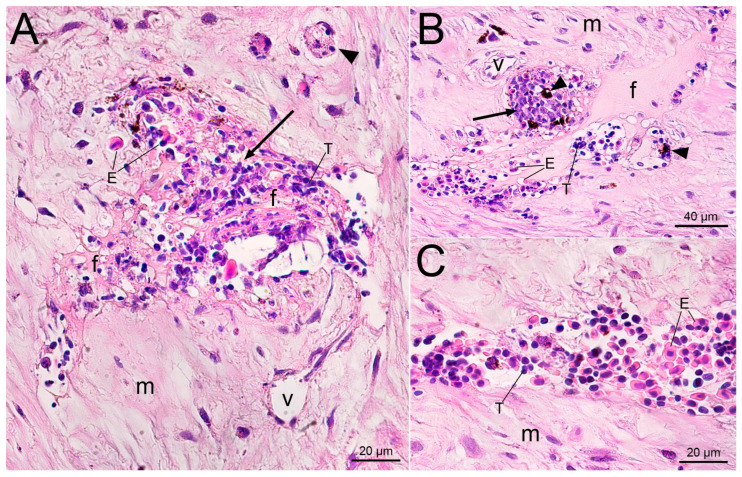
Cross−section of the injection site 3 days after the injection of saline into the trout adipose fin. (**A**) Blood clot (arrow) in the lumen of the injection channel 3 days after the injection. The arrowhead points to the collagen cable perpendicularly crossing the section plane; (**B**) Aggregation of monocytes (arrow) at the injection site, which phagocytize cellular debris. Arrowheads point to melanocytes; (**C**) An enlarged photo of a forming thrombus. Letter designations: E—damaged erythrocytes, T—activated thrombocytes, f—fibrin deposits, v—blood vessels, m—collagen matrix.

The epidermis of the intact adipose fins consisted of 5–10 outer layers of squamous cells alternating with sparse goblet mucous cells resting on a layer of columnar cells attached to the basal lamina (Figure 2C). The deeper dermal stroma comprised regularly arranged bundles of collagen fibers running parallel to the skin surface and hardly distinguishable in the transverse section. The underlying layer of the hypodermis consisted of collagen microfibrils assembled in dense rods (actinotrichia) and in the cross section looked like dense eosinophilic ovals, usually surrounded by fibroblasts. The subcutaneous space was about a third of the thickness of the fin and consisted of a collagen matrix, an amorphous substance of loose collagen fibers, interspersed with stellate fibroblasts (Figure 2B,C). In trout juveniles, the collagen matrix was homogeneous along all sections, with only rare small blood vessels and collagen cables (5–10 micron in diameter) crossing the cut plane perpendicularly (Figure 3A).

The mechanical damage caused by the injection procedure resulted in a rupture of the collagen matrix and blood capillaries and torsion of collagen fibers at the injection site (Figure 3). Three days after saline injection, a thrombus formed inside the lumen of the injection channel, consisting of damaged, pycnotic, or apoptotic erythrocytes and small rounded basophilic cells with a compacted nucleus (nucleus fragments or activated platelets and lymphocytes). The scattered cells of the blood clot were held together by the eosinophilic substance of the fibrin deposits. Due to a weak capillary network in the connective tissue, the hemorrhage caused by trauma was localized in the lumen of the injection channel and was not dispersed in the collagen matrix.

Any tissue damage inevitably leads to the activation of local inflammation to remove fragments of damaged cells, neutralize the possible infection, and stimulate scarring in the wound. In control fish treated with saline, basophilic monocytes with a large pale nucleus, sometimes forming conjunctions, were attracted to the injection site, which indicated the beginning of thrombus resorption on the third day after injury. Pigmented amoeboid melanomacrophages, presumably involved in hemoglobin utilization, have also been observed sporadically in blood cloth.

**Figure 4:**
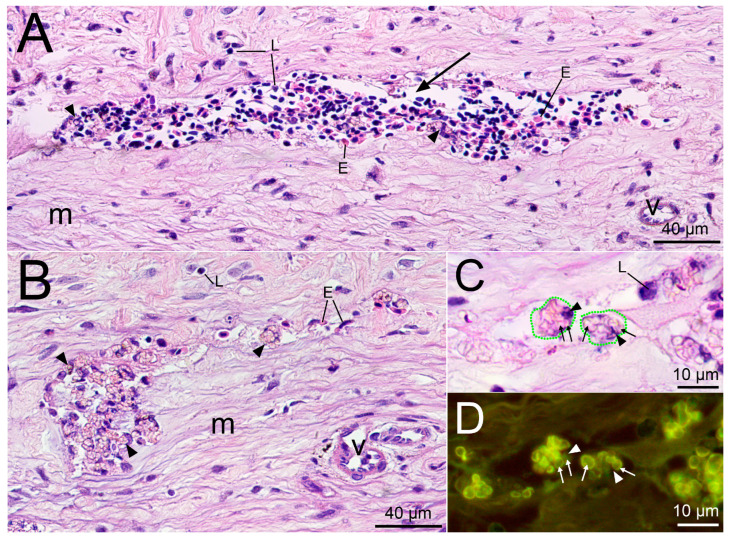
Cross−section of the injection site 3 days after the subcutaneous injection of PMs suspension into the trout adipose fin. (**A**)—The injection channel (arrow) is filled with damaged erythrocytes and leukocytes. Arrowheads point to phagocytes with engulfed microcapsules. (**B**) Aggregation of phagocytes (arrowheads) loaded with polymeric microcapsules at the injection site; (**C**) Enlarged photo of phagocytes (cell boundaries are indicated by the green dotted line) with violet nucleus (arrowheads) and pink spherical microcapsules in the cytoplasm (arrows), bright field microscopy; (**D**) The fluorescent image of panel C depicts the polymeric microcapsules loaded with fluorescent dye. The entire cytoplasmic space is occupied by engulfed microcapsules (arrows), resulting in the nucleus (arrowheads) being pushed to the periphery of the cell. Letter designations: E—damaged erythrocytes, L—leukocytes, v—blood vessels, m—collagen matrix.

A similar picture of local hemostasis and trauma−induced inflammation was observed 3 days after the injection of uncoated PMs (Figure 4). In addition to the leukocytes involved in degradation of erythrocyte and tissue debris, there were many monocytes at the injection site loaded with engulfed microcapsules. The phagocytosis that was observed was very active, so that the PMs almost completely filled the cytoplasm of the immune cells (Figure 4C,D). The free−lying PMs were absent in the wound, which indicates the completion of the process of recognition and phagocytosis of the microparticles by immune cells on the first days after injection. It is important that the examination of histological sections revealed phagocytes loaded with fluorescent PMs in the tissues and vessels of the fin far from the injection channel, demonstrating the active elimination of foreign material from the wound by the third day after injection.

**Figure 5:**
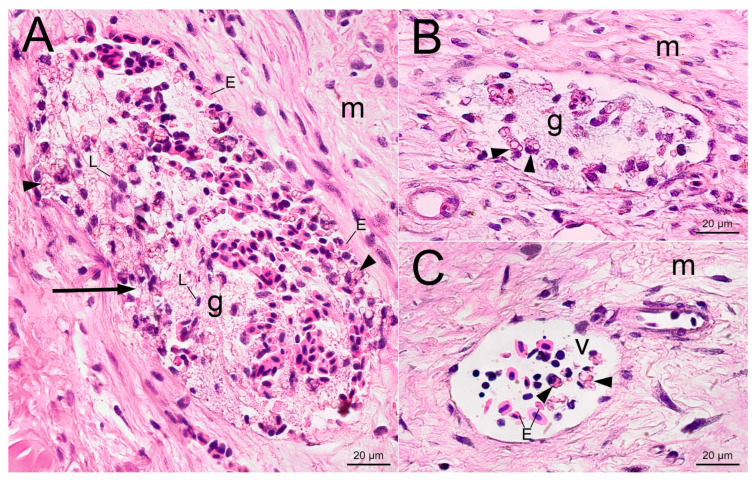
Cross−section of the injection site 3 days after the subcutaneous injection of PMs−PAAH into the trout adipose fin. (**A**) The injection channel (arrow) is filled with 2.5% PAAH (g) with incorporated PMs and spilled erythrocytes. Arrowheads indicate phagocytes with engulfed polymeric microcapsules; (**B**) Hydrogel (g) with phagocytes with engulfed microcapsules (arrowheads); (**C**) Blood vessel (v) with phagocytes with engulfed microcapsules (arrowheads) outside of the site of injection. Letter designations: E—damaged erythrocytes, L—leucocytes, m—collagen matrix.

The dynamics of inflammation after the introduction of 2.5% PAAH with PMs did not significantly differ from that after the administration of PMs alone (Figure 5). On the third day, the injection channel was filled with spilled erythrocytes, attracted leukocytes and microcapsules, which were mainly engulfed by phagocytes and small areas of intact hydrogel. The hydrogel appeared as a purple basophilic network structure with denser and less dense areas, potentially indicating inhomogeneous PAAH polymerization at low monomer concentration. As with the injection of PMs alone, the microcapsules polymerised within the hydrogel were transported from the injection site to the distal tissues by phagocytes; therefore, low−density PAAH did not effectively prevent the penetration of immune cells into the implanted material.

**Figure 6:**
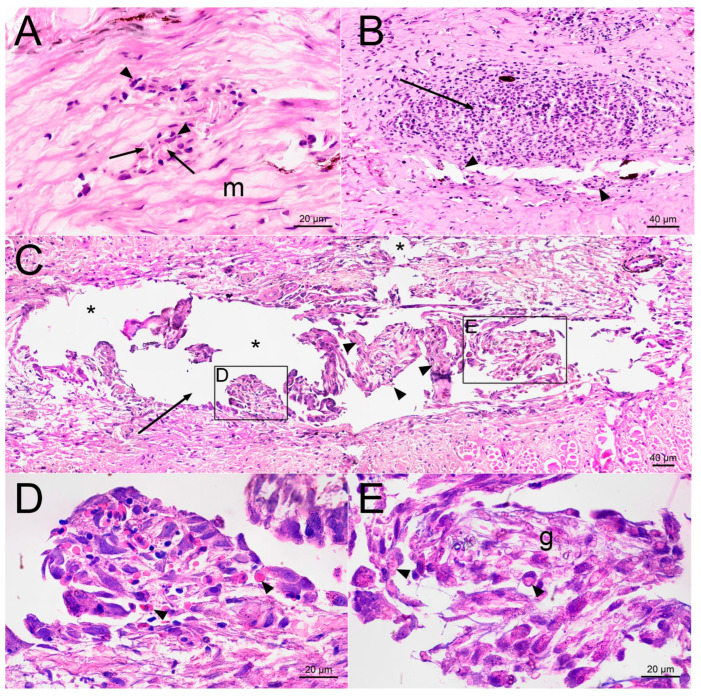
Cross sections of the injection site 14 days after injection into the trout adipose fin. (**A**) Saline injection site with necrotic collagen fibers (arrows) and leukocytes (arrowheads) in the injection channel surrounded by normal collagen matrix (m); (**B**) Site of PMs injection. The injection channel (arrow) is filled with inflammatory cells, including phagocytes engulfing PMs (arrowheads); (**C**) PMs−PAAH injection site. The injection channel (arrow) is filled with nodular aggregates formed by phagocytes that engulf PMs (arrowheads). Asterisks (*) mark the material that fell out of the section (processing artifacts); (**D**) Enlarged photo of panel C showing damaged and apoptotic erythrocytes (arrowheads); (**E**) Enlarged photo of panel C with phagocytes (arrowheads) infiltrating the gel (g) and engulfing PMs.

Two weeks after the injury, the normal attenuation of inflammation was observed in the fin after saline injection (Figure 6A). The edges of the wound were approximated, and the remaining lumen of the injection channel was filled with remnants of necrotic hypercontracted collagen fibers surrounded by phagocytes. No erythrocytes or leukocytes were found in the surrounding collagen matrix, which corresponds to the normal state of the adipose fin tissue. The connective tissue almost entirely restored its integrity, which indicates a successful healing process.

On the contrary, the subcutaneous administration of PMs suspension leads to ongoing inflammation in the adipose fin 14−days post−injection (Figure 6B). Inside the injection channel, there was a homogeneous mushy substance, consisting of disorganized macrophages, mature segmented neutrophils and lymphocytes. No precursors of late phases of healing, such as the development of granulation tissue or the formation of a fibrous membrane around the foreign material were detected in the wound area.

At the end of the second week after semi−liquid PMs−PAAH injection, a chronic inflammatory process was also detected on histological sections of the trout adipose fin (Figure 6C–E). The hydrogel was dissociated and the low density regions were completely lost potentially due to resorption. As a result, the gel did not integrate with the tissue and often fell out of the sections, leaving gaps with uneven, torn edges. Small intact areas remained inside large accumulations of denser PAAH, which indicated the non−free penetration of phagocytes into the gel material. The injection site was edematous, and in the lumen of the injection channel there was an infiltrate scattered among nodular aggregates formed by monocytes and fibroblasts. The observed organization of cells into nodules and lumps may precede the formation of multiple epithelioid granulomas, a normal foreign body isolation reaction, followed by a state of indolent chronic inflammation.

A microscopic study performed 14 days after the administration of PMs, PMs−PAAH or physiological solution revealed no fibrosis, hyalinisation, calcification or necrosis in the adipose fins of the studied fish. The deposits of collagen fibers around the injection channel, which are usually the result of chronic inflammation caused by a foreign body, were weak and confined to places of mechanical disturbance of the regular structure of the connective tissue (creases, folds or breaks) and, presumably, occurred as a result of the classical wound healing process.

## 3. Discussion

Various synthetic non−biodegradable hydrogels are used to enclose microparticles in inert polymeric scaffolds, which are considered biocompatible, although the immune response to the implant can never be reduced to zero [4,6,27]. Previously, we have shown that polyacrylamide, at a monomer concentration of 13%, is able to prevent the destruction of injected polymeric microcapsules by immune cells in vivo for up to at least 9 days [16]. Then, semi−liquid 2.5% PAAH has been proposed as an appropriate carrier for micron−sized sensors due to the ease of fabrication and administration into tissues.

Available data on the histocompatibility of low density 2.5% polyacrylamide are controversial. This gel formulation has a history of use as a cosmetic filler in the 1990s in China, Eastern Europe, and South America (trade names Interfall, Aquamid. etc.); however, there are accumulating data on late complications from its administration to humans (local inflammation, fibrosis and gel migration). The rate and extent of inflammation induced by 2.5% polyacrylamide has been shown to be dependent on injection volume and tissue type [18]. It has been reported that PAAH is generally less amenable to encapsulation and healing in metabolically active tissues than in connective tissues [28,29]. Meanwhile, the time from the introduction of PAAH to the health complaints varied from 6 months to decades [18,20,28,29]. Therefore, according to the data in the literature, this hydrogel can be considered satisfactorily biocompatible for many physiological tests in an animal model lasting for days. At the same time, there are only fragmentary data on the short− and midterm effects of 2.5% PAAH injection on the immune response [17,30].

Conversely, uncovered PMs were shown to have a strong local irritating effect. The polymeric microcapsules were shown to be rapidly engulfed by phagocytes, which transported them to the mucous membranes, presumably to be eliminated from the fish body. Overall, it was demonstrated that, despite their immunogenicity, non−biodegradable (more than 3 weeks) PMs have little potential to cause systemic inflammation or affect distant tissues [31,32].

In this work, we did not reveal the masking effect of a 2.5% polyacrylamide coating on the recognition of polymeric microcapsules (PMs) by the cellular immunity of fish at the time point of 3 days. According to a microscopic analysis of tissue sections, PMs impregnated in low−density PAAH were not retained at the injection site, as they were engulfed and transported by phagocytes on the first days after injection. It is possible that the insufficient number of crosslinks of polyacrylamide chains in a gel with a low monomer concentration makes the polymer 3D network insufficiently coherent and, therefore, the hydrogel was unable to significantly delay the active cross−migration of immune cells. Our study was not designed to assess the structure of the hydrogel or the chemical transformations in vivo, therefore we cannot determine whether the squeezing macrophages pull apart or engulf the polymer chains. Therefore, further studies are needed to investigate the in vivo transformation of the polyacrylamide coating and its potential impact on tissue engraftment or rejection.

At the tissue level, both PMs and PMs−PAAH were highly irritating and provoked inflammation at the injection site for a couple of weeks, suggesting that both formulations interfered with the normal healing process. We have previously recorded acceptable fluorescence spectra within two days after injection of PMs loaded with sensory dye and coated with 2.5% PAAH into the muscles of *Danio rerio*, while a week later we observed the penetration of fish immune cells into the hydrogel, accompanied by degradation of the gel matrix [17]. Here, we show that semi−liquid PAAH does not delay the phagocyte attack on the microimplant in the trout fin, and that the stable signal from microsensors registered in zebrafish muscle in the first days after injection may be caused by the slow migration of immune cells into the forming blood clot.

Despite the local irritating effect, the implantation of microdoses of PMs did not cause a systemic response in the body of fish. The administration of about 2 × 10^6^ of both immunogenic and hydrogel−shielded microcapsules in a volume of about 2 µL did not significantly affect the expression of immune genes in the spleen. This amount of PMs corresponds to the number of fluorescent microcapsules sufficient to record the signal from the shallow layers of fish skin using a fluorescent microscope (0.3–12 × 10^6^ microcapsules depending on the concentration and brightness of the dye) [31,33,34,35]. The obtained results confirmed that the injection of microdoses of the polymeric sensor does not affect the health of fish, but leads to a local shift in physiological parameters in the injection site, making the interpretation of the signal spectrum problematic. Thus, the further in vivo assessment of the long−term systemic effects of 2.5% PMs−PAAH, including the potential neurotoxic effects of unbound acrylamide monomer, was not considered necessary in this study.

In summary, according to our results, implanted semi−liquid PAAH has unsatisfactory stability in vivo for continuous monitoring of the composition of biological fluids for at least several days. This hydrogel formulation is easily injected subcutaneously, imitates the extracellular matrix, integrates into tissues without fibrous encapsulation, and, debatably, has low intrinsic immunogenicity. However, the decisive disadvantage of 2.5% PAAH as a carrier for microimplants is that immune cells can pass through the hydrogel and gain access to the microsensor followed by a prolonged inflammatory reaction at the injection site. Therefore, in terms of the rate of penetration of phagocytes and resorption of the gel, microdoses of synthetic 2.5% polyacrylamide do not provide advantages over biodegradable polymers such as alginate, chitosan, polylactic acid, etc., which are also resorbed over days, but are less toxic and more biocompatible.

## 4. Materials and Methods

### 4.1. Preparation of Microcapsules

Fluorescent polyelectrolyte microcapsules (PMs) were fabricated as described in detail previously [35]. The microcapsules were composed of, in total, 12 layers of oppositely charged polyelectrolytes: poly(allylamine hydrochloride) (#283215; Sigma–Aldrich, St. Louis, MO, USA) and poly(sodium 4−styrenesulfonate) (#243051; St. Louis, MO, USA), and covered by the final 13th layer of poly(L−lysine)−graft−poly (ethylene glycol) co−polymer (#SZ34−67; SuSoS, Dübendorf, Switzerland). Microcapsules were loaded with a conjugate of the fluorescein isothiocyanate with albumin (#FD20S; Sigma–Aldrich, St. Louis, MO, USA; Ex 494 nm, Em 512 nm) as a fluorescent probe. Concentration and size distribution of prepared fluorescent microparticles were determined using a Mikmed−2 fluorescent microscope (LOMO, St. Petersburg, Russia) with a hemocytometer followed by analysis in the Fiji 2.1.0. software [36,37]. Obtained 2.7 ± 0.6 µm microcapsules were washed with saline by multiple cycles of sedimentation−resuspension. The stock solution of the capsules was adjusted to a concentration of 1 million/μL and stored with antibiotic solution (50 µg/mL streptomycin with 50 U/mL penicillin) at 4 °C.

### 4.2. Preparation of Injection Mixtures

All injection mixtures were prepared in a laminar air flow cabinet using autoclaved laboratory plastic. To prepare PMs suspension, 6.4 µL of saline with antibiotic solution (50 µg/mL streptomycin with 50 U/mL penicillin) was mixed in a microtube with 53.2 µL of stock of PMs. The mixture was taken from a test tube with a custom cartridge with plunger and stored at 4 °C until use in the experiment. Custom cartridges were made from modified commercial cartridges Actrapid HM Penfill (Novo Nordisk, Bagsværd, Denmark) for insulin pens, rinsed repeatedly with autoclaved water.

To prepare PMs in polyacrylamide gel (PMs−PAAH), 5 µL of 29% acrylamide solution with 1% N,N′−methylenebisacrylamide was mixed with 53.2 µL of stock of PMs in a microtube. Then, 0.9 µL of 10% ammonium persulfate (APS) and 0.9 µL of 10% tetramethylethylenediamine (TEMED) were added to initiate polymerization. The obtained mixture was immediately taken from a test tube with a custom insulin pen cartridge with a plunger. Then, the cartridge was installed in an orbital Intelli−Mixer RM−1L (ELMI, Riga, Latvia) and rotated for 20 min at 50 rpm to prevent sedimentation of the capsules until complete gel polymerization. The finished gel was stored at 4 °C until use in the experiment. A third modified insulin cartridge was filled with saline with antibiotic solution and stored at 4 °C until use in the experiment.

### 4.3. Animals and Housing

Experimental fish were obtained in June 2021 from a local fish farm (The Republic of Karelia, Russia). Juvenile rainbow trout *Oncorhynchus mykiss* (0+) of average weight 65 ± 20 g were transferred to 170 L tanks (5 fish per tank) filled with tap water and maintained at 9–12 °C by a Hailea HC−250A water chiller. Oxygen saturation was maintained at 8.65–10.5 mg/L through ceramic oxygen diffusers. The required hydrochemistry was maintained by daily replacement of about 18–30% of the water. The concentration of NH_4_^+^, NO_2_^−^ and NO_3_^−^ in the water did not exceed 0.04, 0.1 and 10 mg/L, respectively, and the pH of the water was in the range 6.86–8.53.

### 4.4. Injection into the Adipose Fin

**Figure 7:**
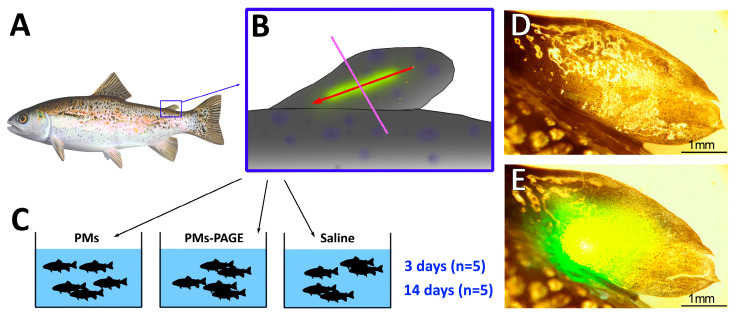
The design of the experiment. (**A**) General anatomy of a trout. The frame represents the adipose fin; (**B**) Scheme of injection. The red arrow indicates the direction of injection and corresponds to the injection tract. Purple line marks the slicing plane; (**C**) Scheme of the experiment; (**D**) The picture shows the adipose fin of a trout after injection of PMs loaded with fluorescent dye; (**E**) The picture shows the fluorescence of an encapsulated dye in the microimplant, excited by a 405 nm laser.

All experimental procedures were conducted in accordance with the EU Directive 2010/63/EU for animal experiments and the Declaration of Helsinki; the protocol of the study was registered and approved before the start of the experiment by the Animal Subjects Research Committee of the Institute of Biology at Irkutsk State University (Protocol #06/2021). On the day of the experiment, the fish were randomly divided into three experimental groups (PMs; PMs−PAAH and saline injected, *n* = 10 in each group, total 30 fish). Animals were individually anesthetized in an emulsion of clove oil in water (0.04 mL/L) until the fish turned over on its side and stopped responding to a light pinch of the fin (about 2 min). The individuals were immobilized on a wet sponge, the adipose fin was rinsed with 50 µg/mL streptomycin with 50 U/mL penicillin solution. Then, using a NovoPen 4 insulin pen (Novo Nordisk, Denmark) with modified insulin cartridges with chosen experimental solution, 2 μL of the mixture was injected along the sagittal plane from distal toward anterior edge of the adipose fin (Figure 7). A disposable insulin needle (29 G, SFM Hospital Products GmbH, Berlin, Germany) was used for each individual. The remains of the injection mixtures were introduced into the solid TBS culture medium and incubated at 37 °C for 48 h to control the microbial contamination of the solutions.

Based on the recommended tolerable doses of polyacrylamide and acrylamide monomer, the prepared PMs−PAAH were used as is, without removal of residual monomers [19]. The 2.5% polyacrylamide concentrations used corresponded to approximately 2.5–25 ppm unbound cytotoxic acrylamide monomers, comparable to the recommended 5 ppm dose for consumer products [19]. At the microdoses of 2 µL of polyacrylamide applied, the resulting exposure was 0.8 µg/kg body weight of fish (residual acrylamide monomer concentration of approximately 0.00008–0.0008 µg/kg body weight). Compared with the safe dose of 0.5 mg/kg bw, which has been reported to cause no systemic adverse effects (including neurotoxicity), this concentration of acrylamide was considered negligible for in vivo tests.

After 3 (*n* = 5) or 14 days (*n* = 5), the fish were euthanized in an emulsion of clove oil in water (0.1 mL/L) with aeration for 2–3 min. Usually within two weeks, the fish immune system has fully developed an acute or chronic response, so that in the absence of infection or other immunogenic factors, the proliferative and remodeling stages of wound healing are observed by this time [38,39,40]. The proximal part of the fin closest to the back was cut off and placed in buffered neutral 10% formalin (Sigma–Aldrich, St. Louis, MO, USA). The abdominal cavity of the fish was opened laterally, and the spleen was removed and weighed. Then, a piece of spleen 1–3 mm wide was cut out and placed in IntactRNA solution.

### 4.5. RNA Extraction and Quantitative PCR (qPCR)

RNA extraction was performed from spleen tissue sections fixed and stored for about a week at 4 °C in IntactRNA (Evrogen, Moscow, Russia) followed by transfer to −80 °C. The tissue was homogenized in 1000 μL TRI Reagent (MRC, Cincinnati, OH, USA) with two 3 mm stainless steel beads (Qiagen, Hilden, Germany) using a TissueLyser LT (Qiagen) with a pre−cooled rotor in three consecutive rounds (50 rpm, 2 min). Then, tissue debris was precipitated by centrifugation (13,680× *g* for 5 min at 4 °C). The supernatant was transferred to a new tube and mixed with chloroform (Vekton; 300 μL chloroform per 1000 μL solution). Phase separation was performed by centrifugation (13,680× *g* for 15 min at 4 °C), and the RNA from the upper aqueous phase was further purified with the RNeasy Mini Kit (Qiagen) according to the instructions of the manufacturer. RNA quantity and integrity were estimated by agarose gel electrophoresis in 1× TAE buffer.

Complementary DNA (cDNA) synthesis was performed with a RevertAid First Strand cDNA synthesis kit (Thermo Scientific, Waltham, MA, USA) with oligod (T) primers (Evrogen) according to the recommendation of the manufacturers, and up to 5% volume of the cDNA synthesis reaction was used as a template for qPCR. qPCR was performed with a StepOne Plus cycler (Applied Biosystems, Waltham, MA, USA) and SensiMix Hi−Rox reagents (Bioline, Memphis, TN, USA) in at least three technical replicates with the following cycling conditions: initial denaturation (95 °C for 10 min) and 40 cycles of denaturation at 95 °C for 15 s, primer annealing at 58 °C for 15 s and elongation at 72 °C for 15 s. The oligonucleotides used for qPCR are listed in Table S1. The absence of non−specific product bands was confirmed with melting curve analysis and agarose electrophoresis. Amplification efficiency was tested and lied within the 90–120% range for all primer pairs. The possibility of amplification from residual genomic DNA was checked with RNA (no reverse transcriptase) controls, and all primer pairs only yielded products with cDNA. The expression of the immune genes was normalized to the level of *EF−1α*, as it has already been demonstrated to be a suitable reference gene for immune gene expression profiling [41,42,43].

### 4.6. Histological and Histopathological Examination

To assess the integrity and immunogenicity of the hydrogel in vivo (and therefore its functionality as an implant cover), tissue organization was analyzed in paraffin−embedded sections of the trout adipose fin. For this, adipose fin samples fixed in 10% neutral formalin were stored for about 2 months, and then the fixative was replaced with 70% ethanol. Paraffin embedding was carried out according to the processing protocol for biopsy specimens (Table S2). Paraffin molds of dehydrated and paraffin infiltrated tissues were serially sectioned at 6 μm thickness with an RWD Minux S700 microtome (RWD Life Science, Shenzhen, China). Five series of sections with intervals of 40 microns were taken from each sample. The obtained slides were examined using a Leica DMLB fluorescence microscope (Leica Microsystems, Wetzlar, Germany) with a ToupCam UCMOS05100KPA camera (ToupTEK Photonics, Hangzhou, China) to detect fluorescence of dye−loaded microcapsules on histological sections. Next, microsections were deparaffinized and stained with hematoxylin (BioVitrum, St. Petersburg, Russia) and eosin (BioVitrum, Russia) with standard staining procedures. The resulting slides were examined by bright field microscopy (Leica Microsystems, Wetzlar, Germany) to investigate morphological changes at the injection site at the tissue and cellular level.

### 4.7. Data Analysis

Morphological parameters and immune gene expression of the fish from the treatment groups were compared by the nonparametric Mann–Whitney test with Benjamini–Hochberg correction for multiple testing using coin [44] and base stats packages for R [45]; *p*−values ≤ 0.05 were considered statistically significant.

## Data Availability

Data are contained within the article or supplementary material.

## Supplementary Materials

The following supporting information can be downloaded at: https://www.mdpi.com/article/10.3390/gels9080629/s1, Table S1: Primers used for gene expression analysis in rainbow trout; Table S2: Paraffin embedding protocol for biopsy specimens used for adipose fin processing.
